# Protective Effect of *Beta*-Carotene against Myeloperoxidase- Mediated Oxidative Stress and Inflammation in Rat Ischemic Brain Injury

**DOI:** 10.3390/antiox11122344

**Published:** 2022-11-27

**Authors:** Hassan N. Althurwi, Rehab F. Abdel-Rahman, Gamal A. Soliman, Hanan A. Ogaly, Faisal K. Alkholifi, Reham M. Abd-Elsalam, Saleh I. Alqasoumi, Maged S. Abdel-Kader

**Affiliations:** 1 Department of Pharmacology, College of Pharmacy, Prince Sattam Bin Abdulaziz University, Al-Kharj 11942, Saudi Arabia; 2 Department of Pharmacology, National Research Centre, Giza 12622, Egypt; 3 Department of Pharmacology, College of Veterinary Medicine, Cairo University, Giza 12613, Egypt; 4 Department of Chemistry, College of Science, King Khalid University, Abha 61421, Saudi Arabia; 5 Department of Biochemistry, College of Veterinary Medicine, Cairo University, Giza 12613, Egypt; 6 Department of Pathology, College of Veterinary Medicine, Cairo University, Giza 12613, Egypt; 7 Department of Ecosystem and Public Health, Faculty of Veterinary Medicine, University of Calgary, Calgary, AB T3R 1J3, Canada; 8 Department of Pharmacognosy, College of Pharmacy, King Saud University, P.O. Box 2457, Riyadh 11451, Saudi Arabia; 9 Department of Pharmacognosy, College of Pharmacy, Prince Sattam Bin Abdulaziz University, Al-Kharj 11942, Saudi Arabia; 10 Department of Pharmacognosy, College of Pharmacy, Alexandria University, Alexandria 21215, Egypt

**Keywords:** neurological deficit, motor coordination, caspase-3, real time PCR

## Abstract

Oxidative stress and inflammatory reaction play critical roles in ischemia/reperfusion (I/R) injury in the brain. β-carotene (βCAR) is a naturally occurring pigment present in fruits and vegetables that expresses antioxidant and anti-inflammatory activities. This study was conducted to investigate the involvement of Bcl2/Bax and NF-κB signaling pathways in the potential protective role of βCAR against brain injury in a middle cerebral artery occlusion (MCAO) rat model. A focal brain ischemia model was created for 2 h, followed by reperfusion. Rats were given 10 and 20 mg/kg of βCAR for 7 days orally before induction of ischemia, at the start of reperfusion, and 3 days after ischemia. Scores of neurological deficit were rated 24 h after induction of ischemia. Motor coordination and spontaneous coordinate activities were assessed using rotarod and activity cage, respectively. After 2 h of the last dose, the animals were killed and their brains were extracted for further examinations. The results of the study show that βCAR diminished the score of neurological deficits and ameliorated motor coordination, balance, and locomotor activity in the I/R control group. Further, βCAR resulted in diminution of malondialdehyde (MDA) and augmentation of reduced glutathione (GSH) contents, as well as the elevation of superoxide dismutase (SOD), glutathione peroxidase (GPx), and catalase (CAT) enzyme activities in the brain homogenates of I/R rats. βCAR treatment significantly reduced nuclear factor kappa B (NF-κB) brain content and myeloperoxidase (MPO) activity and ameliorated the histological alterations in the brain tissues. βCAR significantly suppressed Bcl-2-associated X protein (Bax) and caspase-3 expression, as well as upregulated B-cell lymphoma-2 (Bcl-2) expression, suggesting a neuroprotective potential via downregulating NF-kB and protecting the rat brain against the I/R-associated apoptotic injury.

## 1. Introduction

Ischemic stroke is one of the cerebrovascular illnesses that occur due to insufficient blood circulation to the brain. It is the main cause of cerebrovascular problems worldwide, and it shows high rates of morbidity and mortality among people with the disease [1]. Ischemic stroke can result in the death of neurons, causing behavioural defects, such as defects in sensory motor function, impairment of spatial orientation, and impairment of association learning and memory [2]. Neuronal death caused by cerebral ischemia determines the mortality and disability rate of the disease [3]. Upon cerebral ischemia/reperfusion (I/R), some pathophysiological events, including activation of microglia cells, infiltration of peripheral inflammatory cells, and production of inflammatory mediators, and matrix metalloproteases in the infarct area occur [4]. Consequently, various processes participate in the pathophysiology of ischemic stroke, including inflammation, oxidative stress, and apoptosis. Moreover, stroke causes disruption of the blood–brain barrier, apoptosis, and inflammation [5,6]. Oxidative stress was identified as the most damaging pathological mechanism causing ischemic brain damage. As a result, specific agents of antioxidant effects can be used in cerebral I/R to protect neurons from oxidative stress [7].

Carotenoids are a group of pigments found in animals, higher plants, fungi, and algae with a sequence of colors ranging from yellow to red [8]. Carotenoids are effective antioxidants that reduce oxidative stress [9]. Almost 750 carotenoids with either *E* (*trans*) or *Z* (*cis*) orientations were isolated from natural sources [10]. β-carotene (βCAR) is one of the major carotenoids found in nature. βCAR is an orange-colored, non-oxygenated carotenoid present in yellow-orange fruits and vegetables [11]. Dietary lipids and lipid-soluble vitamins, including βCAR are absorbed in the small intestine and delivered to the peripheral tissues. βCAR concentration in human plasma is a good indicator of the bioavailability of dietary βCAR, representing pro-vitamin A amounts absorbed by the intestinal epithelia. βCAR is stored and metabolized mainly in the liver, followed by adipose tissue, kidney, skin, and lungs [12,13].

βCAR was demonstrated to possess antioxidant activity in vitro [14]. As revealed in a former study, βCAR employs its antioxidant potential via inhibiting the expression of NADPH oxidase subunits, scavenging ROS, and raising the expression/activity of the body antioxidant enzymes [15]. βCAR also quenches free oxygen species, leading to inhibition of lipid peroxidation in addition to immune system activation [16]. Health problems resulting from increased oxidative stress could be controlled by βCAR [14]. Therefore, the goal of the study was to investigate the potential of the neuroprotective effects of the antioxidant; βCAR on the behavioural dysfunction, oxidant/antioxidant imbalance, and histopathological alterations induced by cerebral ischemia/reperfusion (I/R) injury in rats.

## 2. Materials and Methods

### 2.1. Animals

Procedures of the animal study were performed according to the protocol approved by the Ethics Committee of the National Research Centre (NRC), Egypt (MERC-18-086), and the Institutional Animal Care and Use Committee at Cairo University (Approval number: CU-II-F-85-18). Adult male Wistar rats, at five months of age and with an average weight of 150–180 g, were supplied by the Animal House Colony at the NRC, Egypt. A few days before the experiment, rats were housed in typical environmental settings (12 h cycles of darkness and light, 22–25 °C, and 40%–60% relative humidity). The study was complied with the regulations of the ethics committee of the NRC, which follows the National Regulations on Animal Welfare and Institutional Animal Ethical Committee. The number of animals were kept as minimum as possible to perform suitable statistical analysis in order to minimize animal suffering.

### 2.2. Drugs

β-carotene (βCAR) was purchased from Arab Co. for Pharmaceuticals and Medicinal Plants (MEPACO-MEDIFOOD)- Egypt; (*Beta*-Carotene Forte, B. No. 1200820, molecular weight: 536.87).

### 2.3. Induction of Focal Cerebral I/R Injury

The middle cerebral artery occlusion (MCAO) was applied to rats for 2 h to induce focal cerebral I/R damage followed by reperfusion [17]. Animals were anesthetized using ketamine (100 mg/kg) and xylazine (10 mg/kg) anesthesia and were fixed in the supine position on a surgery board. In the left carotid region a midline cervical incision was performed to expose the external carotid and the common carotid arteries. A non-traumatic microvascular clamp was inserted from the carotid bifurcation into the internal carotid artery, resulting in a blocking of the origin of the middle cerebral artery. Two hours after the blocking, clamps were removed to start reperfusion. The cervical incision was then sutured and the rats were allowed to recover. Rectal temperature of the rats during the operation were maintained at 37 °C by heating lamp. Sham control rats were exposed to the same procedure except for MCAO.

### 2.4. Experimental Protocol

Rats were allocated into four groups, each comprising 12 animals. The 1st group (sham group) was composed of animals subjected to similar surgical procedures, except for artery occlusion, and was given liquid paraffin (the vehicle). The 2nd group was assigned as the I/R control group and treated with the vehicle. The 3rd and 4th groups were assigned as I/R + βCAR at 10 mg/kg (βCAR-10) and 20 mg/kg (βCAR-20), respectively [18]. Liquid paraffin and βCAR were orally administered by intragastric tube for 7 days prior to artery occlusion at the initiation of reperfusion and continued for 3 days after artery occlusion.

Twenty-four hours after MCAO surgery, neurological deficit scores were assessed in all rats. Afterward, animals from each group (*n* = 12) were divided into two subgroups of six animals each. The 1st subgroup was subjected to rotarod testing and biochemical evaluation, while the 2nd one was tested using the activity cage, neuronal cell count, histopathological investigation, as well as gene expression analysis.

### 2.5. Evaluation of Neurological Deficit

After 24 h of MCAO surgery, neurological deficit scores were observed in rats after 24 h of artery occlusion by personnel blinded to the animals’ treatment using a 5-grade score [19]. Scoring manner was: grade 0, absence of neurological deficit; grade 1, inability to fully extend the contralateral anterior limb; grade 2, the circles when pulled by its tail but takes a normal position when resting; grade 3, spontaneous circling; grade 4, absence of spontaneous movement and a low level of consciousness; and grade 5, animals’ death. Rather than sham-operated rats, rats scoring either 0 or 5 were excluded from the study.

### 2.6. Behavioural Evaluation

#### 2.6.1. Evaluation of Motor Coordination and Balance

Motor coordination and balance were monitored using a rotarod device (Model No. 7750; Ugo Basile) following the procedures of Vijitruth et al. [20]. Before MCAO, rats were trained individually on an accelerating rotating rod of an accelerating speed level between 4 and 40 rpm. Stable baseline values were obtained from three separate trials for each animal at 5 min durations and a cut off time of 300 s. Upon testing, each rat was placed individually on the rod revolving from 4 to 40 rpm for over 5 min. Each rat was checked 3 times with 10 min duration. In each trial, an observer who was blinded to the experimental groups recorded the time the rats fell for a maximum time of 300 s. For each rat, the mean of the three trials was calculated. Passive rotation along the rod without walking was accounted as a fall.

#### 2.6.2. Evaluation of Spontaneous Motor Activity

Measurement of the spontaneous motor activity of the rats was achieved using a grid floor activity cage (Model No. 7430, Ugo-Basile, Comerio, Italy). Movements of the rat interrupted the infrared rays and were automatically recorded. The ray interference information was processed by the instrument software giving the horizontal motor activities. Rats were adapted to the test room for 1 h before the MCAO surgery. Basal activity counts were recorded for each rat for a 5 min session individually in the activity cage. At the end of the session, rats were softly returned back to their home cage. Between sessions the grid floor was cleaned using 70% (*v*/*v*) alcohol solution in distilled water to exclude any olfactory signals. At the testing time, similar to the training sessions, each animal was observed in the activity cage for 5 min and the final activity counts were recorded [21].

### 2.7. Euthanasia and Brain Tissue Samplings

All rats were sacrificed by cervical decapitation using sharp scissors after 2 h of the last dose of βCAR, and their brains were excised.

### 2.8. Biochemical Analyses

Fresh brain tissues were collected and carefully washed with chilled saline. Tissue homogenate (10% *w*/*v*) in 0.1 M phosphate buffer (pH 7.4) was prepared for each brain tissue. The homogenates were subjected to centrifugation at 10,000× *g* at 4 °C for 15 min by the help of cooling centrifuge. The supernatants were separated, immediately frozen using liquid nitrogen, and stored at −80 °C until further examination.

#### 2.8.1. Measurement of Brain Glutathione (GSH) Content

Reduced glutathione content in the brain homogenate was determined following the reported methods [22,23]. Briefly, 0.5 mL of the brain homogenate supernatant was mixed with 0.5 mL 10% trichloro-acetic acid. The mixture was centrifuged at 1800× *g*/5 min and 4 °C. From the supernatant, 0.1 mL was mixed with 1.8 mL of phosphate buffer pH 8.0 in a clean test tube. Next, 0.1 mL of Ellman reagent was added. After 5 min, the absorbance was measured at 412 nm against a blank containing distilled water instead of the sample. Reduced glutathione levels were calculated using the extinction coefficient of 1.36 × 10^4^ M^−1^ cm^−1^ and the results are expressed in μmol GSH/g tissue [24].

#### 2.8.2. Assessment of Lipid Peroxidation (LPO) in Brain Tissues

The quantitative estimation of lipid peroxidation in the brain tissues of rats was performed as previously reported [25]. From the homogenate supernatants, 0.5 mL were mixed with 4.5 mL of TCA-TBA reagent composed of 20% TCA, and 0.8% TBA, 3:1. The mixtures were heated in a water bath for 20 min. The mixtures were cooled then centrifuged at 3000× *g* for 10 min. The resulted supernatants were collected, and the absorbances were measured against blank at 535 nm. The amounts of MDA generated were calculated, using the molar absorption coefficient of 1.56 × 10^5^ M^−1^ cm^−1^ and presented as nmol/g tissue [24].

#### 2.8.3. Assessment of Superoxide Dismutase (SOD) Activity in Brain Tissues

SOD activity was assessed in the brain tissues at 420 nm as formerly described [26]. From the brain supernatants, 100 μL were added to 0.1 M Tris-HCl buffer pH 8 containing 30 μmol/L catalase and 24 mmol/L pyrogallol in a net volume of 3 mL.

#### 2.8.4. Determination of Glutathione Peroxidase (GPx), Catalase (CAT), Nuclear Factor Kappa B (NF-κB), and Myeloperoxidase (MPO) in Brain Tissues

The activities of GPx, CAT, and MPO, and the level of NF-κB were estimated in the brain tissues following the instructions of the manufacturer using a commercial ELISA kit, Kamiya Biomedical Co., WA, USA.

### 2.9. Histopathological Evaluation

Left brain lobes were obtained from all groups and maintained in 10% neutral buffered formalin for 24 h. Brain specimens were processed to obtain 4–5 μm paraffin embedded sections. Brain sections were stained with hematoxylin and eosin (H and E). The brain cortex and hippocampus were examined under light microscope to perform the brain lesion scoring according to the parameter described earlier [27]. The infarct areas were determined, and the ratio of the infarct area to the whole slide area was calculated and represented as percentage of the ischemic areas by using a Lieca Qwin 500 Image Analyzer (Leica, Cambridge, England).

### 2.10. Neuronal Cell Count

Sections of the left brain lobe were dehydrated and deparaffinized using xylene and then stained with toluidine blue stain [28]. The number of unharmed neuronal cells in correlation to the total number of the neuronal cells was determined under high magnification power (×400). The percentage of intact cells was calculated in both brain cortex and hippocampus using the ImageJ analyzer (software v. 1.41).

### 2.11. Immunohistochemistry of Caspase-3

The tissue specimens of all groups were deparaffinized and rehydrated. The antigen retrieval was carried out using 10 mM citrate buffer, pH 6.0 according to the reported method [29]. Mouse monoclonal anti-caspase-3 antibody (sc-56053, Santa Cruz Biotech, Dallas, TX, USA) at 1:50 was added to tissue specimens and incubated overnight in humid space. In negative control slides the primary antisera were omitted with 1 mg/mL BSA (Sigma, Kawasaki, Japan). The tissue sections were thoroughly washed with Tris-buffered saline to remove unconjugated antibodies. After that, the sections were incubated for 10 min with a biotinylated goat anti rabbit and mouse antibody (Thermo Fisher scientific, Waltham, MA, USA), thoroughly washed with TBS, and then DAB (3,3 diaminobenzidine) was added to the sections. Finally, the sections were counterstained with Mayer’s hematoxylin and mounted with DPX. The photographed pictures were analyzed using ImageJ. The percentage of positive immune-staining cells was calculated in 7 microscopic fields/groups in both the brain cortex and hippocampus.

### 2.12. Gene Expression Analysis by Real Time PCR

The expression of antiapoptotic B-cell lymphoma-2 (Bcl-2) and proapoptotic Bcl-2-associated X protein (Bax) mRNA in the brain tissues of the rats were evaluated by RT-PCR following the I/R of the brain. Total RNA was extracted using the total RNA extraction kit (Thermo Fisher Scientific, Inc.) as required by the manufacturer. After genomic DNA removal using DNase I, one microgram total RNA was subjected to reverse transcription for cDNA synthesis using Superscript II reverse transcriptase (Thermo Fisher Scientific) according to the manufacturer’s instructions. The mRNA expression levels were quantified by the real-time fluorescence PCR method, and the β-actin gene was used as an internal reference to standardize the different transcription values [29]. Each PCR reaction was performed in triplicate. The specificity of the PCR amplification was confirmed by melting curve analysis at the dissociation stage. The comparative expression of Bax and Bcl-2 mRNA were calculated after normalization to β-actin based on the cycle threshold (CT) formula F = 2−ΔΔCT, ΔΔCT = (the average CT value of target genes in the treated group—average CT value of β-actin in the treated group) − (average CT value of target gene in normal control group—average CT value of β-actin in normal control group). The primer base sequences (5′-3′) used in this study were: Bax upstream, ACCAAGAAGCTGAGCGAGTG; Bax downstream, CCAGTTGAAGTTGCCGTCTG; Bcl-2 upstream, GAGGATTGTGGCCTTCTTTG; Bcl-2 downstream, CGTTATCCTGGATCCAGGTG; β-actin upstream, ATGGTGGGTATGGGTCAG; and β-actin downstream, CAATGCCGTGTTCAATGG [30].

### 2.13. Statistical Analysis

Data are presented as the mean ±SE for groups of 6 rats in both the behavioural tests and the biochemical tests. The comparisons between groups were achieved using two-way analysis of variance (ANOVA), followed by Tukey’s multiple comparisons test. Data analyses were performed with Graph Pad Prism v. 5.0 (Graph Pad Software, Inc., CA, USA). Data with *p* ≤ 0.05 were considered statistically significant.

## 3. Results

### 3.1. Evaluation of Neurological Deficit

**Figure 1:**
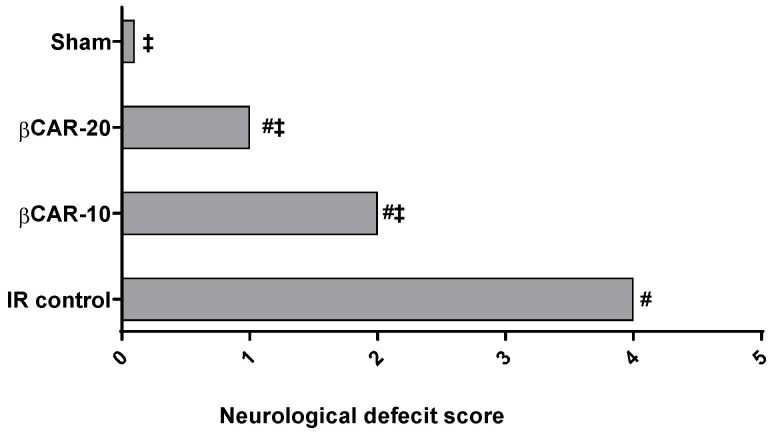
Neurological deficit scores 24 h after induction of cerebral I/R. #, ‡ denotes significant difference at *p* ≤ 0.05 vs. the sham and I/R control group, respectively, (*n* = 12).

Neurological deficits were evaluated 24 h after reperfusion (Figure 1). Sham-controll rats revealed no neurological deficits; however, the I/R control group exhibited the uppermost neurological deficit score supporting the success of the surgical procedure and brain ischemia. Neurological deficit scores for βCAR-10 and βCAR-20 groups were significantly less (2.25 ± 0.19 and 1.50 ± 1.07, respectively) than that of the I/R control group (3.92 ± 0.22).

### 3.2. Behavioural Evaluation

#### 3.2.1. Evaluation of Motor Coordination and Balance

**Figure 2:**
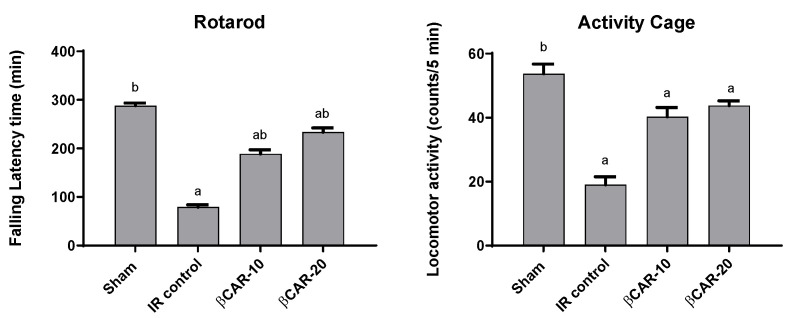
Effect of βCAR on motor coordination (rotarod) and locomotor activity (activity cage) in I/R control rats. ^a, b^ denotes significant difference at *p* ≤ 0.05 against sham and I/R control groups, respectively, (*n* = 6).

Two days after middle cerebral artery occlusion (MCAO) surgery, evaluation of motor coordination and balance was conducted in rats (Figure 2). I/R injury resulted in cerebral ischemia of the left brain hemisphere, evidenced by significant decrease in the final falling latency time by 72.5% as compared to sham-operated rats. The obtained results show that βCAR at 10 and 20 mg/kg significantly enhanced the motor coordination ability of MCAO rats as compared to the I/R control group. Rats of βCAR-10 and βCAR-20 groups exhibited a significant increase in the final falling latency time of 138.3% and 194.9% compared with the I/R control rats, respectively.

#### 3.2.2. Evaluation of Spontaneous Motor Activity

I/R control rats displayed a significant decrease in their locomotor activity by 64.6% in comparison with sham-operated rats (Figure 2). βCAR at 10 and 20 mg/kg resulted in a marked increase in the locomotor activity of rats by approximately 2.12 and 2.31 folds, respectively, compared with the I/R control group

At a 0.05 level of significance, there is a significant difference between the sham and I/R control groups.

### 3.3. Effect of βCAR on Oxidative Stress Biomarkers

As depicted in Table 1, the content of non-enzymatic antioxidants, such as glutathione (GSH), and the activities of enzymatic antioxidants, such as catalase (CAT, glutathione peroxidase (GPx) and superoxide dismutase (SOD), were significantly diminished in the brain of I/R control group as compared to sham-operated rats (*p* ≤ 0.05). While malondialdehyde (MDA) level was significantly elevated in the I/R control group relative to sham rats. Administration of βCAR at 10 and 20 mg/kg significantly boosted GSH and SOD levels and increased GPx, and CAT activities when compared to the I/R control group. Brain contents of MDA were significantly restored to normal level in βCAR-10 and βCAR-20 groups.

**Figure 3:**
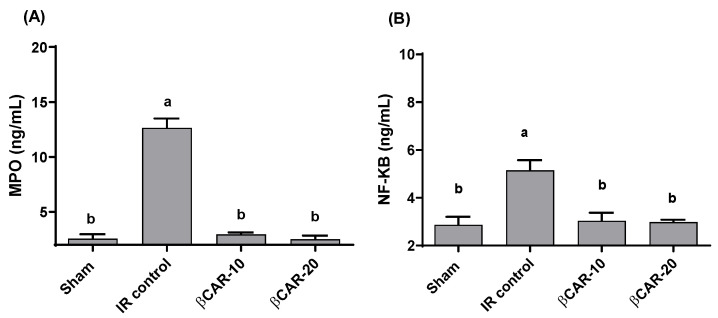
Effect of βCAR on MPO (**A**) and NF-κB (**B**) in brains of rats with focal cerebral ischemia-reperfusion. ^a, b^ denotes significant difference at *p* ≤ 0.05 against sham and I/R control groups, respectively, (*n* = 6).

Myeloperoxidase (MPO) activity and NF-κB content in the brain were significantly (*p* ≤ 0.05) elevated in I/R rats (12.6 ± 0.85 and 5.15 ± 0.422 ng/mL, respectively) as compared to sham-operated rats (Figure 3). I/R-rats treated with βCAR at 10 and 20 mg/kg showed significant decrease in the MPO activities (76.7% and 80.2%, respectively) and nuclear factor kappa B (NF-κB) levels (41.1% and 42.1%, respectively) as compared to I/R- rats.

### 3.4. Histopathology of the Brain

**Figure 4:**
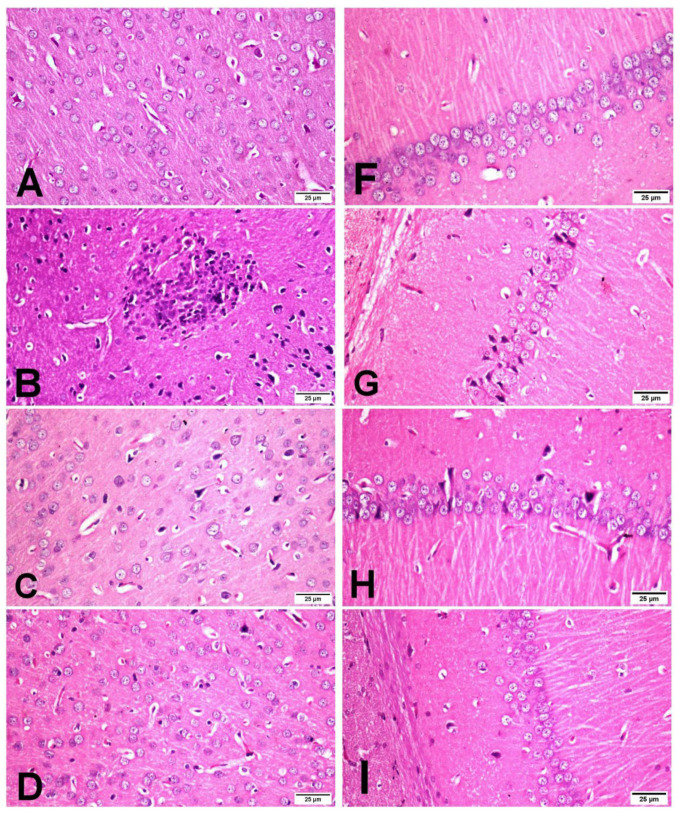
Effect of *beta*-carotene on histopathology and neuronal ischemic damage in cerebral cortex and hippocampus areas in focal cerebral ischemia-reperfusion model ×400. (**A**–**D**) Cerebral cortex. (**F**,**G**) Hippocampus. (**A**) Sham rats showing normal histological findings of the cerebral cortex. (**B**) I/R control group showing focal area of coagulative necrosis with gliosis and the surrounding neuron show neuronal degeneration. (**C**) βCAR-10 and (**D**) βCAR-20 groups showing marked reduction in the number of degenerated neurons. (**F**) Sham group showing normal histologic picture of brain hippocampus. (**G**) I/R control group showing pyramidal cell loss with numerous pyramidal cell necrosis. (**H**,**I**) βCAR-10 and βCAR-20 groups showing marked reduction in pyramidal cell necrosis and loss. Scale bar 25 μm.

**Figure 5:**
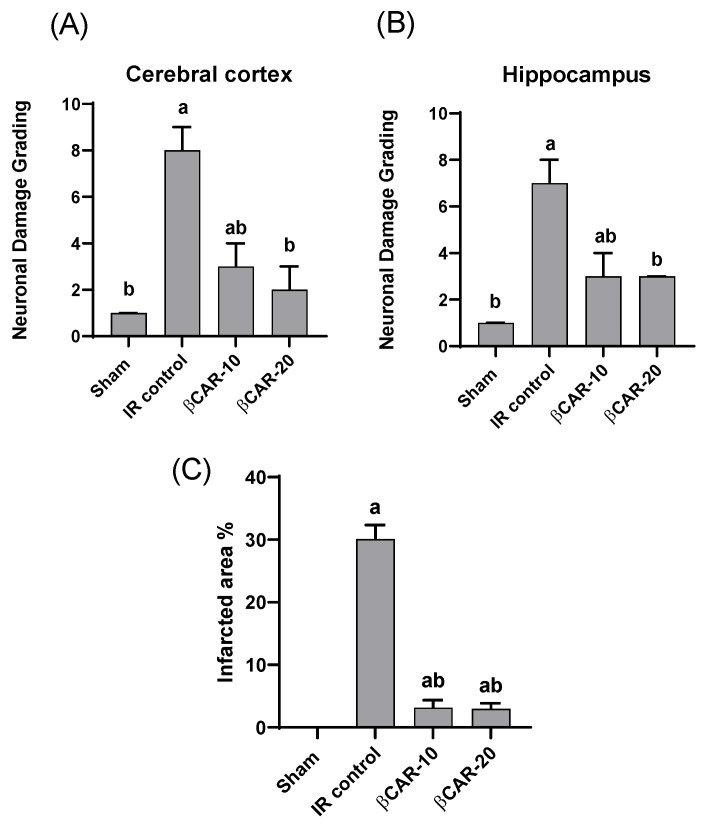
Effect of *beta*-carotene on ischemic neuronal grading and infarcted area %. (**A**) Ischemic neuronal grading in cerebral cortex. (**B**) Ischemic neuronal grading in hippocampus, *n* = 6, values expressed as median with interquartile changes, ^a, b^ denotes significant difference from sham and I/R control groups, respectively, at *p* ≤ 0.05. (**C**) Effect of *beta*-carotene on infarcted area % in focal cerebral ischemia-reperfusion model. Infarcted area % represented as ratio of the infarct area to the whole slide area. *n* = 6, values expressed as mean with interquartile changes, ^a, b^ denotes significant difference at *p* ≤ 0.05 against sham and I/R control groups, respectively.

The cerebral cortex and hippocampus of the sham group revealed normal histological findings with intact neuronal and pyramidal cells (Figure 4A,F). The ischemic control group showed multiple areas of coagulative necrosis with gliosis, neuronal degeneration with neurophagia, astrogliosis, microgliosis, and proliferation of endothelial lining blood vessels in grey matter (Figure 4B); while white matter revealed axonal fragmentation with demyelination of nerve fibers. Moreover, there were massive numbers of degenerated and necrosed pyramidal cells with neurophagia in the hippocampus, especially in cornu ammonis (CA1) (Figure 4G). However, βCAR-10 and βCAR-20 groups showed negligible histopathological changes in the form of few numbers of degenerated and necrosed neurons, mild gliosis, and single pyramidal cell necrosis in CA1 in the hippocampus region (Figure 4C,D,H,I). Regarding the brain lesion scoring, the ischemic control group exhibited a significant elevation in all parameters compared to the sham group. The βCAR-10 and βCAR-20 group revealed a significant decrease in such parameters when compared with the ischemic control group (Figure 5A,B). In addition, the percentage of infarct area was significantly reduced in βCAR-10 and βCAR-20 groups compared to the ischemic control group (Figure 5C).

### 3.5. The Neuronal Cell Count

**Figure 6:**
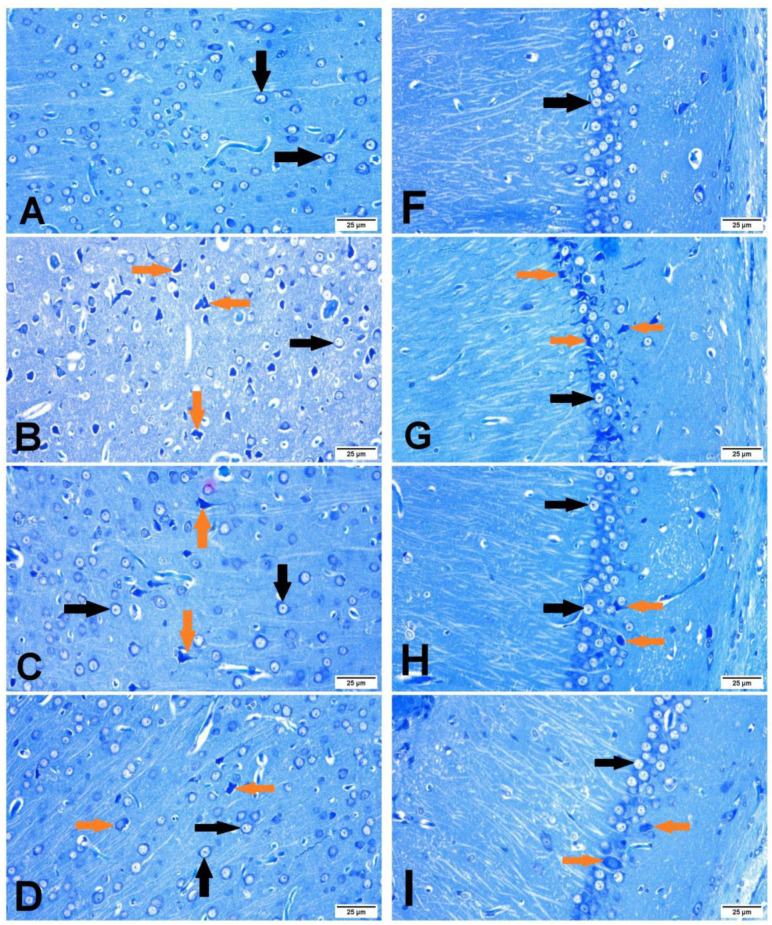
Brain sections stained with toluidine blue, showing the effect of beta-carotene on neuronal cell count in cerebral cortex and hippocampus region in focal cerebral ischemia-reperfusion model ×400. Intraneuronal cell (black arrows) and non-intact neuronal cell (green arrows). (**A**–**D**) Cerebral cortex. (**F**–**I**) Hippocampus. (**A**,**F**) Sham group. (**B**,**G**) I/R control group. (**C**,**H**) βCAR-10. (**D**,**I**) βCAR-20 group. Scale bar 25 µm.

**Figure 7:**
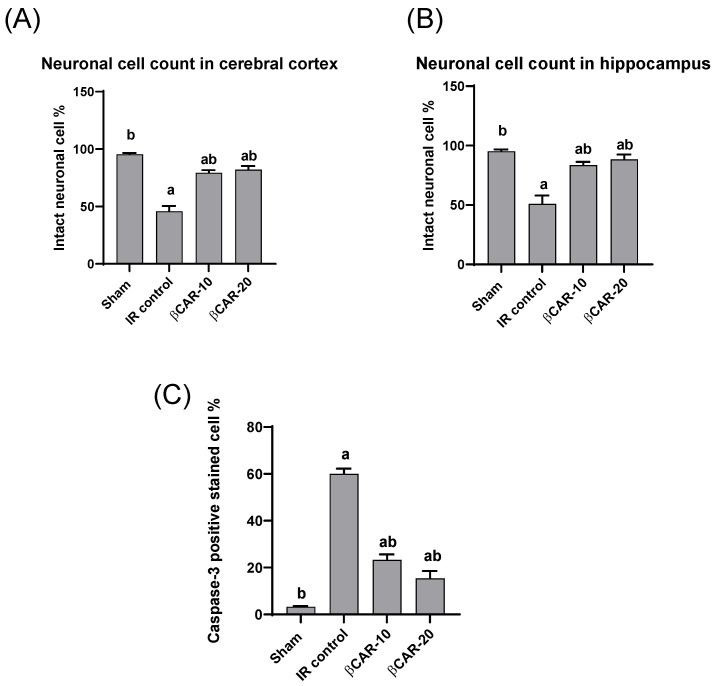
Effect of *beta*-carotene on neuronal cell count and caspase-3 protein expression. (**A**,**B**) The bar chart represents the percentage of intact neuronal cells in the cerebral cortex and hippocampus, respectively. (**C**) The bar chart represents the percentage of caspase-3 immunopositive cell in the cerebral cortex. All values are expressed as mean ± SE (*n* = 6). ^a, b^ denotes significant difference at *p* ≤ 0.05 against sham and I/R control groups, respectively.

The histopathological examination of the brain sections stained with toluidine blue revealed that normal neuronal cell appeared intact with clear vesicular nucleus, while non-intact neuronal cell appeared shrunken and darkly stained (Figure 6). The I/R control group showed a large number of non-intact neuronal cells and revealed a significant reduction in the percentage of normal intact neuronal cells in both the cerebral cortex and hippocampus when compared with sham group. The βCAR-10 and βCAR-20-treated groups exhibited significant increase in the percentage of normal intact neuronal cells in both areas (cerebral cortex and hippocampus) compared to the I/R control group (Figure 7A,B).

### 3.6. Immunohistochemistry of Caspase-3

**Figure 8:**
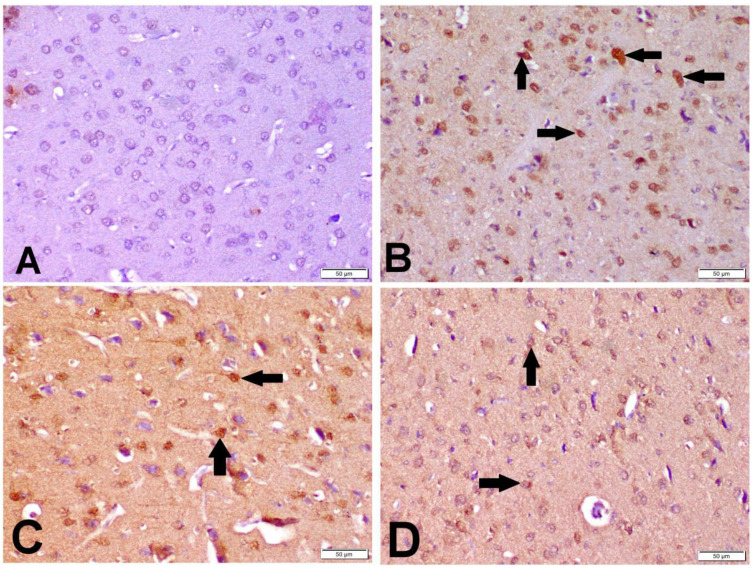
Effect of *beta*-carotene on caspase-3 protein expression in cerebral cortex of focal cerebral ischemia-reperfusion model ×200. Caspase-3 immunopositive cell was with dark brown cytoplasmic and nuclear color (arrows). (**A**) Sham group, (**B**) I/R control group, (**C**) βCAR-10 group, and (**D**) βCAR-20 group. Scale bar 50 µm.

The cytoplasm and nucleus of the neurons and glial cells both displayed caspase-3 immune-positive staining. Ischemic control rats expressed significant caspase-3 immune-positive cell percentage as compared with the sham control group; while the percentage of caspase-3 immune-positive cells was significantly lower in the I/R + βCAR-10 and I/R + βCAR-20 groups compared to the ischemic control rats (Figure 7C and Figure 8).

### 3.7. Expression of Bax and Bcl-2 mRNA in Brain Tissues

**Figure 9:**
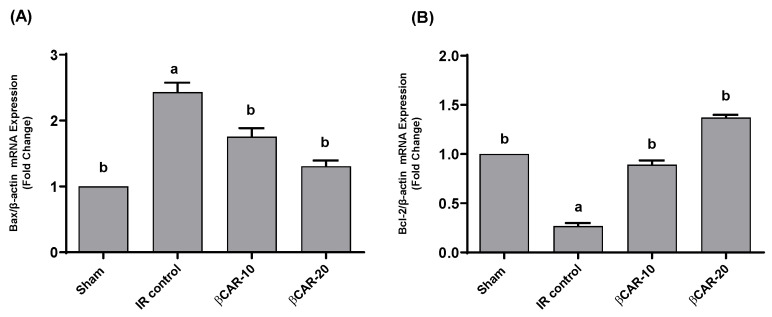
Effects of βCAR on Bax (**A**) and Bcl-2 (**B**) mRNA expression in ischemic cerebral tissues of rats. Values are presented as fold changes of sham control value ± SEM (*n* = 6) after normalization by β-actin. ^a, b^ denotes significant difference at *p* ≤ 0.05 against sham and I/R control groups, respectively.

In the current study, we used RT-PCR to compare the mRNA expression of the apoptosis-promoting Bax and the apoptosis-inhibiting Bcl2 in the βCAR-10 and βCAR-20 groups (*n* = 6) to their expression levels in the sham operation group (*n* = 6) and the I/R control group (*n* = 6). When compared to the sham group, the mRNA expression level of Bax in the ischemic side of the brain of the I/R model rats tended to increase (*p* ≤ 0.05); while there was a decreased trend (*p* ≤ 0.05) in the expression of Bax in the rats’ ischemic brain tissue in the βCAR-10 and βCAR-20 groups (Figure 9).

Compared to the sham group, the expression of Bcl-2 was downregulated in the ischemic brain of I/R control group (*p* ≤ 0.05). The expression of Bcl-2 in the βCAR-10 and βCAR-20 groups tended to be upregulated (*p* ≤ 0.05) in comparison to the model group (Figure 9). The Bax/Bcl2 ratio in βCAR-10 and βCAR-20 was significantly increased compared to I/R control ratio, indicating the antiapoptotic effect of βCAR (Table 2). The results obtained demonstrate that the Bax/Bcl-2 ratio is significantly higher in the I/R control rats compared to sham-operated rats. In ischemic rats pre-treated with βCAR-10 and βCAR-20, the Bax/Bcl-2 ratio was significantly decreased compared to the ischemia group (Table 2).

## 4. Discussion

Middle cerebral artery occlusion (MCAO) is the foremost common cause of ischemic stroke, with high death rates of 40%–80% [31]. The great majority of cerebral stroke cases are brought on by the temporary or permanent blockage of a cerebral blood vessel, which eventually causes disruptions in brain functions and can result in either reversible or permanent neurological deficits. Further, post-reperfusion lesion arises as a result of excessive production of reactive oxygen species (ROS), resulting in apoptotic neuronal death, inflammation, and oxidative damage [32,33].

The hippocampus and basal forebrain are the main parts of the brain involved in memory and learning processes. Cognitive impairment could arise when distortion in the function or structure of these brain areas occur [34]. In the current study, the left cerebral I/R model gave rise to increased neurological deficit scores in rats. This finding is supported by previous reports where the neurological deficit score was elevated in the I/R control rats compared to the sham animals [21,35]. Further, neurological deficit scores were significantly lowered in βCAR-10 and βCAR-20 groups compared to the I/R control rats.

In a previous study, βCAR demonstrated marked improvement in the neurological function and reduced brain injury after traumatic brain injury in mouse models. Moreover, βCAR treatment significantly decreased cell death and increased neuronal survival in the peri-injury region [36]. Additionally, a decrease in locomotor activity and motor incoordination in I/R rats was recorded in the current study as compared to the sham group. Pretreatment with either doses of βCAR for 7 days significantly increased the locomotor activity using the activity cage device, and motor incoordination using the rotarod device after 24 h of reperfusion compared to the I/R control animals. These findings are consistent with the previous reports by Hattori et al. [37,38]. In a mouse model of streptozotocin-induced Alzheimer’s disease, βCAR attenuated cognitive deficit through its anti-oxidative efficacy, suppression of acetylcholinesterase, and the reduction in amyloid β-protein fragments [39].

In response to I/R injury, apoptosis, necrosis, impaired microvascular function, and edema elicited were observed. Overproduction of ROS as a result of oxidative stress during brain I/R is a major cause of brain damage [40]. Estimation of oxidative stress biomarkers is crucial to assess the mechanisms of action of emerging neuroprotective agents in the brain tissues of ischemic rats. In I/R rats, brain levels of GSH and SOD, as well as the activities of GPx and CAT, were significantly reduced compared to the sham-operated group. Moreover, brain contents of MDA and the activity of MPO and NF-κB were elevated considerably in I/R rats as compared to sham-operated rats (*p* ≤ 0.05).

The antioxidant properties of various diets and the potential for the prevention of various chronic diseases are attributed to various carotenoids found in those foods. The correlation between plasma carotenoid concentrations and the incidence of stroke was studied extensively in cross-sectional and case-control studies. Denoting that plasma antioxidant activity might be an imperative factor in the neurological protection against damage caused by stroke-associated oxidative stress [41]. βCAR revealed its ability to act against lipid peroxidation and reduce oxidative stress, and is consequently considered as a significant protective factor in brain-related diseases [39].

In ischemic stroke, myeloperoxidase (MPO) activation plays a vital role in brain oxidative damage. The elevated MPO activity was testified in both patients with ischemic stroke and in experimental animal models [42,43]. Thus, MPO inhibition might offer a promising therapeutic target for stroke therapy via attenuating oxidative damage and neuroinflammation in ischemic stroke [44].

It is well recognized that the NF-κB transcription factor has a key function in regulation of inflammatory responses, immune functions, and cell survival by acting as a central mediator for induction of multiple pro-inflammatory genes [45]. Consequently, the deregulated activation of NF-κB signaling contributes to the pathogenesis of various inflammatory processes, including cerebral ischemic damage. NF-kB triggers multiple genes involved in the ischemic inflammatory reactions, such as TNF-α, IL-6, IL-1β, COX-2, and iNOS [45]. Neuronal NF-κB could be activated by many different stimuli including inflammatory mediators, brain-derived growth factors (BDNF), neuronal growth factors (NGF), and excitatory neurotransmitters [45,46]. Emerging studies indicate that excessive ROS production in cerebral I/R is associated with dysregulated NF-κB activity leading to neuronal cell death [47].

Considerable evidence suggests that neuronal NF-κB signaling disruption contributes to neurodegeneration and neuronal death after cerebral ischemia [48]. Moreover, activated NF-κB in glial cells, which triggers the excessive production of ROS and pro-inflammatory cytokines that aggravate secondary neurotoxicity and neuronal damage [45]. Previous studies revealed an association between NF-κB activity and the severity of stroke [49]. In the same context, NF-κB inhibition revealed a reduction in stroke size and neuro-deficit in the cerebral ischemic injury model [50]. This suggests that agents that can suppress NF-κB will counteract the damaging mechanism of ischemic stroke.

Accumulating evidence suggests that targeting the NF-kB signaling pathway could be employed as a therapeutic strategy in the treatment of cerebral I/R injury [51,52]. Consistent with these findings, the current study demonstrated that βCAR suppressed NF-κB activity in the ischemic brain tissues, suggesting that the inhibitory effects on NF-κB and its downstream pro-inflammatory mediators may be one of the crucial mechanisms of βCAR against cerebral I/R injury.

βCAR is an efficient quencher of free oxygen species, able to prevent oxidative stress and lessen neuronal injury [41,53]. These properties delineated the inhibiting ability of βCAR against oxidative stress-induced inflammation via suppressing pro-inflammatory adipokines. According to various in vitro and in vivo models, the antioxidant properties of the carotenoids may be responsible for their anti-inflammatory action. The antioxidant effects of carotenoids are anticipated to protect the brain against oxidative damages.

In the current work, brain sections from the I/R control group stained with toluidine blue revealed a large number of non-intact neuronal cells with significant reduction in the percentage of normal intact neuronal cells in both the cerebral cortex and hippocampus when compared with the sham group. However, brain sections from βCAR-10 and βCAR-20-treated groups exhibited a significant increase in the percentage of normal intact neuronal cells in the cerebral cortex and hippocampus compared to the I/R control group. Moreover, the I/R control group displayed a significant elevation in the percentage of caspase-3 immunepositive cells when compared with the sham-operated group. Meanwhile, the βCAR-10 group and βCAR-20 revealed a significant decrease in the percentage of caspase-3 immune-positive cells compared to the I/R control group.

It is well established that caspases play an essential role during apoptotic cell death. Precisely, caspase-3 is crucial during neuronal development and under pathological conditions such as cerebral ischemia. It is the most abundant cysteine aspartase expressed in the adult rodent brain. Moreover, activated caspase-3 subunits are detected in ischemic tissues by immunoblotting and immunohistochemistry [54]. Consistent with our findings, astaxanthin, a dietary carotenoid that is a structurally similar to βCAR, showed a substantial decrease in cerebral infarction and caspase-3 activity with an increase in post-stroke locomotor activity after MCAO in rats [55].

Notably, neuronal apoptosis associated with cerebral I/R is a multifactorial process in which oxidative stress, pro-inflammatory mediators, caspase signaling, calcium dysregulation, and excitotoxicity are the major factors [56]. Increased cellular activation of Bax and suppression of Bcl2 were reported in several ischemic injuries, supporting the idea that the proapoptotic/anti-apoptotic proteins ratio can determine the cellular fate after cellular response to toxic signals. Following ischemia, activated Bax induces the upregulation of caspase-9 in the brain tissue, which leads to cleavage and activation of caspase-3, the key mediator of apoptosis [57].

Additionally, the anti-apoptotic Bcl2 protein acts as a survival factor in the intrinsic apoptotic pathway through inhibiting and preventing the nuclear translocation of the apoptosis-initiating factor with the subsequent inhibition of caspase cleavage.

The present study demonstrates that βCAR exhibits neuroprotective effects on cerebral ischemia by modulating the expression of Bax and Bcl2 proteins. The obtained results indicate that βCAR may ameliorate cerebral I/R injury by downregulating Bax and upregulating Bcl-2 mRNA expression, thus decreasing the Bax/Bcl2 ratio and inhibiting apoptosis. In addition, our findings prove that restoring Bax/Bcl-2 ratio with βCAR pretreatment effectively regulates the downstream caspase-driven apoptosis after cerebral I/R injury [58].

## 5. Conclusions

In conclusion, βCAR revealed protective benefits against ischemia-induced neurotoxicity *in vivo*. The proposed mechanism of protection involves reducing cerebral edema, suppressing oxidative stress, and preventing apoptosis, suggesting that βCAR can be used as an adjuvant agent in the treatment of ischemic stroke. Additionally, the anti-inflammatory properties of βCAR are mediated via downregulation of NF-kB, caspase-3, and Bax, as well as upregulation of Bcl-2 expression, which protects against neuronal cell death.

## Figures and Tables

**Table 1:** Effect of βCAR on oxidative stress biomarkers in cerebral I/R rats.

	GSH (μmol/g)	SOD (U/g)	MDA (nmol/g)	GPx (U/g)	CAT (U/g)
**Sham**	3.3 ± 0.11 ^b^	87.4 ± 0.3 ^b^	14.0 ± 0.89 ^b^	302.0 ± 19.73 ^b^	2.79 ± 0.061 ^b^
**I/R control**	1.7 ± 0.07 ^a^	52.5 ± 2.9 ^a^	30.6 ± 2.12 ^a^	150.3 ± 8.43 ^a^	0.58 ± 0.026 ^a^
**βCAR-10**	2.7 ± 0.09 ^ab^	65.8 ± 4.06 ^ab^	19.8 ± 1.42 ^ab^	230.4 ± 7.61 ^ab^	1.91 ± 0.023 ^ab^
**βCAR-20**	3.1 ± 0.13 ^b^	69.2 ± 2.12 ^ab^	16.0 ± 1.07 ^b^	245.9 ± 11.8 ^ab^	2.31 ± 0.068 ^ab^

Data are represented as mean ± S.E. of 6 rats per group. ^a, b^ denotes significant difference from sham and I/R control groups, respectively, at *p* ≤ 0.05.

**Table 2:** Bax/Bcl2 mRNA ratio in ischemic cerebral tissues of rats.

	Bax/Bcl2 Ratio
**Sham**	1
**I/R control**	9.07 ^a^
**βCAR-10**	1.97 ^b^
**βCAR-20**	0.95 ^b^

^a, b^ denotes significant difference at *p* ≤ 0.05 against sham and I/R control groups, respectively, *n* = 6.

## Data Availability

Not applicable.
